# The updated role of exosomal proteins in the diagnosis, prognosis, and treatment of cancer

**DOI:** 10.1038/s12276-022-00855-4

**Published:** 2022-09-22

**Authors:** Xinyi Wang, Jing Huang, Wenjie Chen, Genpeng Li, Zhihui Li, Jianyong Lei

**Affiliations:** Thyroid Surgery Center, West China Hospital of Sichuan University, Chengdu, China

**Keywords:** Protein-protein interaction networks, Cancer microenvironment

## Abstract

Exosomes are vesicles encompassed by a lipid bilayer that are released by various living cells. Exosomal proteins are encapsulated within the membrane or embedded on the surface. As an important type of exosome cargo, exosomal proteins can reflect the physiological status of the parent cell and play an essential role in cell–cell communication. Exosomal proteins can regulate tumor development, including tumor-related immune regulation, microenvironment reconstruction, angiogenesis, epithelial–mesenchymal transition, metastasis, etc. The features of exosomal proteins can provide insight into exosome generation, targeting, and biological function and are potential sources of markers for cancer diagnosis, prognosis, and treatment. Here, we summarize the effects of exosomal proteins on cancer biology, the latest progress in the application of exosomal proteins in cancer diagnosis and prognosis, and the potential contribution of exosomal proteins in cancer therapeutics and vaccines.

## Cancer: exploring the potential of exosomes

Exosomes, membrane-bound vesicles released by some cells, contain a cargo of proteins and other biomolecules that can be analyzed to assist the diagnosis of cancer or predict its likely outcome, and may become useful in treating cancer. Zhihui Li, Jianyong Lei, and colleagues at Sichuan University, Chengdu, China, review progress in cancer-focused exosome research. The proteins in exosomes can act as indicators of the health or disease status of cells. They can also control the development of tumors and wider physiological processes that support tumor growth and spread. The authors consider the specific significance of exosome proteins in each of a wide range of common human cancers, and discuss some technical challenges that must be overcome to fully exploit the potential of exosomes to diagnose, monitor, and perhaps treat cancer.

## Introduction

Exosomes arise from multivesicular bodies (MVBs) and are cup-shaped under an electron microscope, with a diameter ranging from 50 to 150 nm. Exosomes are naturally secreted by all kinds of cells and are commonly detected in bodily fluids, including blood, urine, ascites, and saliva^1^. Exosome cargo includes nucleic acids, proteins, lipids, enzymes, and metabolites that indicate the physiological condition of the parent cell^2^. Exosomes have been suggested to play a role in cell–cell communication by direct fusion with the cell membrane and through endocytic pathways and ligand–receptor interactions^3^.

Tumor-derived (TD) exosomes are released by tumor cells and carry substances that can reflect the features of the parental tumor cell^4^. Therefore, exosomes can be applied as tumor diagnostic markers. As an important form of communication between tumor cells and nontumor cells within the microenvironment, exosomes can play an important role in different stages of tumor development, including tumor-related immune regulation, microenvironment reconstruction, angiogenesis, invasion, and distant metastasis^3,5^. The expression profile of exosomal proteins is often significantly different in different types and stages of cancer, indicating that these proteins are closely associated with cancer development and progression^6^. Moreover, other functions of TD exosomes cannot be ignored, including their involvement in restricting immune regulation and enhancing chemoresistance by eliminating chemotherapeutic drugs^7–9^, which might promote primary tumor growth and metastasis.

By examining special subpopulations of exosomes, the cell of origin can be determined based on the increased specificity of exosome cargo. The miRNA content in exosomes is the highest among all kinds of RNAs, and miRNA is stable because it is not easily degraded by RNA enzymes^10^. miRNA has become a preferred molecule in the study of exosomes due to easy enrichment and sampling^11^. The mRNAs in exosomes carry abundant genetic information on tumor cells, so the detection of a specific mRNA in exosomes can be used not only to diagnose cancer and evaluate tumor progression but also to monitor the treatment response^12^. Currently, studies on lncRNAs as tumor biomarkers or prognostic indicators are still in the initial stage, but exosomal lncRNAs are easy to extract and stable in the environment, favorable characteristics for further research^13^. Compared with other exosome cargo, exosomal proteins have the following advantages in cancer diagnostics: (1) exosomal proteins are stable in exosomes and have a long half-life^14^. Moreover, exosomal proteins can act directly on target cells, whereas nucleic acids must be transcribed or translated before exerting activity. Therefore, quantitative and qualitative data on exosomal proteins can provide more accurate information than data on other cargo. (2) Compared with other exosome cargo, exosomal surface proteins can be detected in a small sample volume after a relatively simple isolation procedure. Exosomal proteins can provide abundant, stable, sensitive^15–23^, and unique information^24–27^ (Table 1). (3) Due to advances in mass spectrometry-based detection technology and the continuous optimization of data collection and analysis methods, the protein coverage and sensitivity of comparative proteomics have improved, and specific exosomal proteins in urine from patients with cancer can be detected^14^. The application of proteomics tools to analyze posttranslational modifications in exosome research has also increased the depth of exosome proteomics data in the context of cancer pathogenesis, function, and disease associations^28^. In recent years, researchers have paid increasing attention to exosomal proteins, and numerous studies on exosomal proteins have been recently published. However, few reviews have focused on the function of exosomal proteins in the development, diagnosis, progression, and treatment of cancer^29^. In this review, we summarize the effects of exosomal proteins on tumor development, highlight the diagnostic and prognostic roles of exosomal proteins in various cancers, and illustrate the potential application of exosomal proteins in cancer therapy. The aim of this review is to provide a comprehensive understanding of the biological mechanisms and clinical value of exosomal proteins, deepen understanding of the roles of exosomal proteins in tumorigenesis and cancer progression, and provide suggestions for further application and investigation.

**Table 1:** The diagnostic efficiency of exosome cargo.

Study	Fluid	Cancer type	Patients	Molecule(s) analyzed	Cargo type	Diagnostic efficiency
Su et al.^15^	Serum	Ovarian cancer	50 OC patients, 50 healthy volunteers, and 50 benign ovarian tumor patients	miR-375, miR-1307	miRNA	miR-375 (sensitivity: 61.76%; specificity: 87.88%), miR-1307 (sensitivity: 33.33%; specificity: 94.29%)
Meng et al.^16^	Serum	Ovarian cancer	163 epithelial ovarian cancer (EOC) patients	miR-373, miR-200a, miR-200b and miR-200c	miRNA	miR-200a (sensitivity: 83.9%; specificity: 90%), miR-200b (sensitivity: 52.8%; specificity: 100%) and miR-200c (sensitivity: 31.1%; specificity: 100%)
Jiao et al.^17^	Serum	Hepatoblastoma	89 children with HB	miRNA-34	miRNA	Sensitivity: 94.36%; specificity: 78.30%
Tang et al.^18^	Serum	Colorectal cancer	34 patients with metastatic CRC and 108 with non‐metastatic CRC	miR-320d	miRNA	Sensitivity: 62.0%; specificity: 64.7%
Shi et al.^19^	Cerebrospinal fluid	Glioblastoma	70 glioblastoma patients	miRNA-21	miRNA	The area under curve (AUC) for exosomal miR-21 was 0.927 (95% CI: 0.865–0.985); the AUC of tissue miR-21 for discriminating II/III/IV grade or III/IV grade was 0.751–0.872
Zhao et al.^20^	Serum	Gastric cancer	126 GC patients and 120 healthy people	HOTTP	lncRNA	The AUC for exosomal HOTTIP was 0.827
Ism et al.^21^	Urine	Prostate cancer	30 patients with PC and 49 patients with benign prostatic hyperplasia (BPH)	lncRNA-p21	lncRNA	Sensitivity: 67%; specificity: 52%
Liu et al.^22^	Serum	Laryngeal squamous cell carcinoma (LSCC)	52 LSCC patients and 49 patients with vocal cord polyps	HOTAIR	lncRNA	Sensitivity: 94.2%; specificity: 73.5%
Zheng et al.^23^	Urine	Bladder cancer (BC)	104 BC patients and 104 healthy controls	PCAT-1 and MALAT1	lncRNA	PCAT-1 (sensitivity: 72.1%; specificity: 84.6%) and MALAT1 (sensitivity: 72.1%; specificity: 81.7%)
Pan et al.^24^	Serum	Colorectal cancer	135 CRC patients, 35 patients with benign intestinal diseases (BIDs) and 45 healthy controls (HCs)	hsa-circ-4771	circRNAs	Sensitivity: 54.29%; specificity: 68.57%
Allenson et al.^25^	Plasma	Pancreatic ductal adenocarcinoma (PDAC)	68 PDAC and 54 healthy controls	KRAS	DNA	Mutations detected in 7.4%, 66.7%, 80%, and 85% of controls, localized, locally advanced, and metastatic PDAC patients
Möhrmann et al.^27^	Plasma	Colorectal, melanoma, and non-small cell lung cancer	43 patients progressing to advanced cancer	BRAFV600, KRASG12/G13, and EGFRexon19delL858R	DNA	Mutations in EV DNA corresponding to those in tissue DNA were found in 95% of cases.
Castellanos-Rizaldos et al.^26^	Serum	Non-small cell lung cancer	Training and test cohorts each with 51 mutation-positive and 54 mutation-negative samples	EGFRT790M	DNA	Training: 81% sensitivity, 95% specificity. Test: 92% sensitivity, 89% specificity
Buscail et al.^126^	Peripheral and portal blood	Pancreatic cancer	22 patients with resectable PDAC and 28 controls without cancer	GPC1	Protein	Sensitivity: 100%; specificity: 100%.
Øverbye et al.^92^	Urine	Prostate cancer	16 prostate cancer patients and 15 healthy controls	A diagnosis model of 17 exosomal proteins	Protein	17 proteins showed sensitivities above 60% at 100% specificity, and TM256 had the highest sensitivity (94%)
Moon et al.^87^	Plasma	Breast cancer	Healthy controls (*n* = 81) and patients with breast cancer (*n* = 269)	Del-1	Protein	AUC: 0.961; sensitivity: 94.70%; specificity: 86.36%
Yoon et al.^108^	Serum	Gastric cancer	500 patients with gastric cancer, including 360 with advanced gastric cancer (AGC) and 140 with early gastric cancer (EGC)	GKN1	Protein	Sensitivity: 91.2%; specificity: 96.0%

## Proteins on the surface of exosomes

Exosomal proteins are either encapsulated within the lumen or embedded on the surface of exosomes, which enables the subtyping of exosomes based on surface biomarkers without destroying their structure^30^. Specifically, some proteins (e.g., CD63, TSG101, and Alix) have been recognized as biomarkers for exosomes derived from various cells, while other proteins (e.g., Calnexin) function as negative markers for exosome identification^31^. Exosomes secreted by cells undergoing pathological processes present distinct compositions that serve as markers of their states. Some proteins (e.g., EGFR, EphA2, and EpCAM) on the surface of exosomes are increasingly used to distinguish TD exosomes from nontumor-derived exosomes^6^. Metastatic ovarian cancer (OC) cells can release numerous exosomes that carry E-cadherin, an inducer of angiogenesis^32^. The expression levels of exosomal lipopolysaccharide-binding proteins and E-cadherin were utilized to identify non-small cell lung cancer (NSCLC) and OC cells with metastatic phenotypes^33^. Metastatic melanoma cells secrete exosomes that carry programmed death ligand-1 (PD-L1), which can bind programmed death-1 (PD-1) on T cells to drive immune checkpoint responses^34^. In patients with head and neck squamous cell carcinoma, the levels of exosomal PD-L1 are correlated with disease progression, UICC stage, and lymph node invasion (*P* = 0.0008)^35^. Another study showed that the detection of PD-L1-positive exosomes in blood samples from patients with pancreatic ductal adenocarcinoma was associated with worse survival^36^. Pretreating glioblastoma-derived exosomes with anti-PD-1 antibodies reversed the suppression of T cells and prevented cancer progression^37^. Chen et al.^34^ demonstrated that PD-L1 on the surface of metastatic melanoma-derived exosomes can suppress CD8+ T cells and promote tumor proliferation, and these processes were affected by anti-PD-1 antibody treatment. Exosomal surface proteins can provide sufficient diagnostic and prognostic information for cancers and can be used to monitor treatment responses. Moreover, exosomal surface proteins may provide insight into the mechanisms of exosome biogenesis^38–43^, targeting^44,45^, and interactions^46–49^. We have summarized the main types of exosomal surface proteins and their functions in Table 2.

**Table 2:** Exosomal surface proteins and their roles.

Exosomal proteins	Roles	References
Major histocompatibility complex (MHC): MHC class I, MHC class II	Antigen presentation to induce an immune response	^45^
Tetraspanins (CD9, CD63, CD37, CD81, CD82, CD53)	Protein scaffolding and anchoring in cellular membranes. CD9, CD63, and CD81 are present at high levels in exosomes, are often used as exosome biomarkers, and can influence exosome biogenesis and composition	^39,40^
GTPase, Annexins, Flotillin, Rab GTPases	Crucial in intracellular vesicle transport, including endosome recycling and MVB trafficking to lysosomes. They can mediate intraluminal vesicle budding and tethering of MVBs to the plasma membrane	^42–44^
Glycoproteins (β-galactosidase, O-linked glycans, N-linked glycans)	Specifically interact with receptors and enable the specificity of exosome targeting	^46,47^
Fas ligand, TNF receptor, Transferrin receptor	Exosome targeting and signaling, including the induction of apoptosis and iron transport	^48,49^
Integrin-α, integrin-β, P-selectin	Mediate the interaction, attachment, and membrane fusion with the target cell	^46,75^

## The effect of exosomal protein cargo on cancer biology

### Exosomal proteins and angiogenesis

Angiogenesis is mediated by vascular endothelial growth factor (VEGF), a proangiogenic factor released by endothelial and tumor cells^50^. Exosomes carrying VEGF may be crucial for early tumor angiogenesis^51^. Feng et al.^52^ found that exosomes can induce angiogenesis in MDA-MB-231 cells by activating a specific form of VEGF called 90 kDa VEGF (VEGF_90K_). These researchers also found that Hsp90 localized near exosomal VEGF and decreased the efficiency of bevacizumab^53^. These data indicate that VEGF-activated Hsp90 can promote tumor cell viability. Another research team reported that pancreatic cancer (PC)-derived exosomes can drive angiogenesis by triggering gene expression in human umbilical vein endothelial cells (HUVECs)^54^. The high levels of phospho-Akt and phospho-ERK1/2 in HUVECs demonstrated the angiogenic function of PC-derived exosomes. Other exosomal proteins that are recognized to facilitate angiogenesis and are potential targets of antiangiogenic drugs include carbonic anhydrase 9^55^, annexin II^53^, and WNT4^56^. Taken together, these studies have shown that exosomal proteins correlate with angiogenesis and vascular permeability, thereby promoting cancer progression.

### Exosomal proteins and epithelial–mesenchymal transition

In cancer, epithelial–mesenchymal transition (EMT) is a specific process in which epithelial cells transform into mesenchymal cells^57^. There is a relationship between mesenchymal cell-derived exosomes and EMT in epithelial cells^58^. Simpson et al.^59^ evaluated intracellular and extracellular protein alterations during EMT (in secretomes, cell membranes, and exosomes) by using EMT models of MDCK (kidney epithelial) cells and H-Ras-transformed MDCK (21D1) cells. Proteomic analyses of the secretomes of MDCK and 21D1 cells showed the remodeling of extracellular matrix (ECM) proteins, including decreased levels of basic membrane components (e.g., collagen type IV) and proteases (e.g., matrix metalloproteinase 1 (MMP-1))^60^. Proteomic analyses of 21D1 cell-derived exosomes showed a decrease in some epithelial biomarkers (e.g., E-cadherin and EpCAM) but an increase in mesenchymal biomarkers (e.g., vimentin) and proteinases (e.g., MMP-1)^61^. Therefore, a thorough recognition of the function of exosomal proteins in EMT is needed. Furthermore, therapeutic windows can be exploited during EMT, potentially via pharmacological methods that target exosome biogenesis and secretion mechanisms.

### Exosomal proteins and the tumor microenvironment

**Fig. 1:**
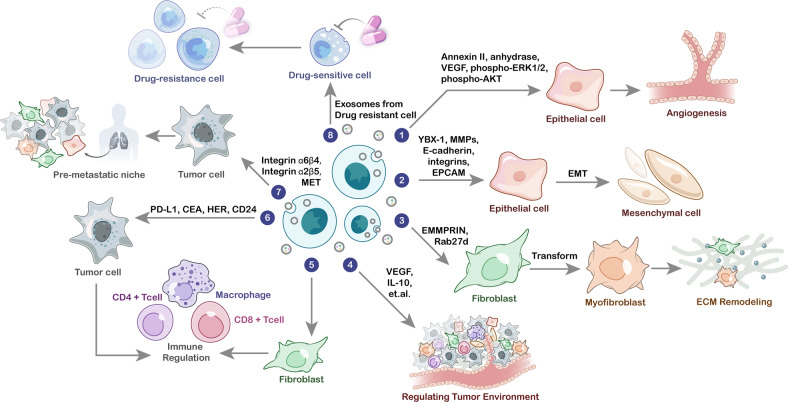
The role of exosomal proteins in cancer biology. Exosomal proteins play an important role in different stages of tumor development, including angiogenesis, epithelial–mesenchymal transition (EMT), extracellular matrix (ECM) remodeling, tumor-related immune regulation, premetastatic behavior, and therapeutic resistance.

Studies have shown that tumor cells can interact with various types of noncancer cells within the tumor microenvironment (TME)^62^. The ECM is considered an important part of the TME and serves as a physical and biochemical support for tumor cells to regulate their function^63^. ECM remodeling can cause quantitative and qualitative alterations in the ECM, which facilitates tumor cell proliferation and metastasis^64^. As a component of the extracellular environment, tissue-derived exosomes interact with the ECM, and this interaction is an important factor determining the biological effect of these exosomes^65^. The ability of tissue-derived exosomes to carry tumor promoters and initiate ECM remodeling in the TME is critical for the survival of tumor cells. Exosomal proteins (e.g., extracellular matrix metalloproteinase inducer) have been found to induce ECM remodeling by activating the generation of MMPs in fibroblasts^66^. MMPs can remodel the ECM by disassembling it and releasing the embedded growth factors, thereby facilitating tumor growth and metastasis. Another study showed that introducing Rab27b into ovarian carcinoma cells can lead to the secretion of exosomes that upregulating MMP2 expression^67^. In addition to their effects on ECM remodeling, exosomal proteins can also influence the TME by immune regulation. He et al.^68^ identified a novel function of endoplasmic reticulum (ER) stress-associated exosomes in mediating macrophage cytokine (i.e., IL-10) secretion in the liver cancer microenvironment and indicated the potential to treat liver cancer by targeting an ER stress-exosomal-STAT3 pathway. Hodgkin lymphoma-derived exosomes can facilitate the transformation of normal fibroblasts into tumor-specific fibroblasts, which causes the release of proinflammatory cytokines (e.g., IL-1α, IL-6, and TNF-α), growth factors (e.g., G-CSF and GM-CSF) and proangiogenic factors (e.g., VEGF)^28,69^. This finding is supported by research on metastatic melanoma-derived exosomes with surface expression of PD-L1^12^. Stimulation with interferon-γ may increase the amount of PD-L1 on the surface of exosomes to suppress the function of CD8^+^ T cells and promote tumor cell proliferation^70^. Therefore, in summary, exosomal proteins have variable effects on immune regulation that alter the microenvironment (Fig. 1).

### Exosomal proteins and premetastatic behavior

Recently, exosomes have been reported to prime distant organs toward a favorable microenvironment (i.e., premetastatic niche), which promotes the survival and proliferation of tumor cells^71^. Peinado et al.^72^ reported that MET was needed for the premetastatic behavior of primary tumors as a result of the exosome-mediated education of bone marrow cells. The expression of Rab proteins, a family involved in exosome generation, was increased in melanoma cells, while silencing RAB27A led to decreased exosome generation, tumor growth, and metastasis^73^. Costa-Silva et al.^74^ reported that exosomal macrophage migration inhibitory factor (MIF) induced the secretion of TGF-β from Kupffer cells, which facilitates the biogenesis of fibronectin in hepatic stellate cells. Therefore, fibronectin then traps and confines macrophages and neutrophils in the liver, promoting the construction of premetastatic niches. Hoshino et al.^75^ found that exosomes released from tumor cells can specifically target recipient cells and prime premetastatic niches in different organs (such as the lung, liver, and brain). In particular, targeting the integrins α6β4 and αvβ5 can reduce exosome interactions and promote lung and liver metastasis, demonstrating that exosomal integrins are involved in predicting organ-specific metastasis. These studies prove that tumor cells can prime target organs by secreting exosomes carrying specific proteins.

### Exosomal proteins and drug resistance

Exosome-mediated multidrug resistance is also an important mechanism of cancer development^9^. Mesenchymal stem cell-derived exosomes have been reported to suppress immune activity and promote chemoresistance in gastric cancer (GC)^76,77^. The underlying mechanism involves the exosome-mediated activation of calcium-dependent protein kinases and the Raf/MEK/ERK cascade in GC cells^78^. Drug resistance-related proteins (e.g., LRP and MRP) have been shown to be regulated by mesenchymal stem cell-derived exosomes and to impact the efficacy of 5-fluorouracil and cisplatin^79^. Tumor cells can also transfer drug efflux pumps to promote drug resistance. P-Glycoprotein^80^, MDR-1^81^, and ATP-binding cassette subfamily B member 1^8^ are drug efflux pumps that transmit multidrug resistance in prostate cancer, OC, leukemia, and osteosarcoma. The capacity of exosomes to transmit multidrug resistance from an adriamycin-resistant variant of MCF-7 to an adriamycin-sensitive variant of MCF-7 was achieved by controlling P-glycoprotein expression^82^. The overexpression of P-glycoprotein is regarded as a potential mechanism of drug resistance that affects the movement of anticancer agents and immunosuppressants. Exosomal proteins have been recognized as important mediators of drug resistance, and further studies have demonstrated the application of exosomal proteins to cancer therapy.

## The diagnostic value of exosomal proteins in human cancer

**Fig. 2:**
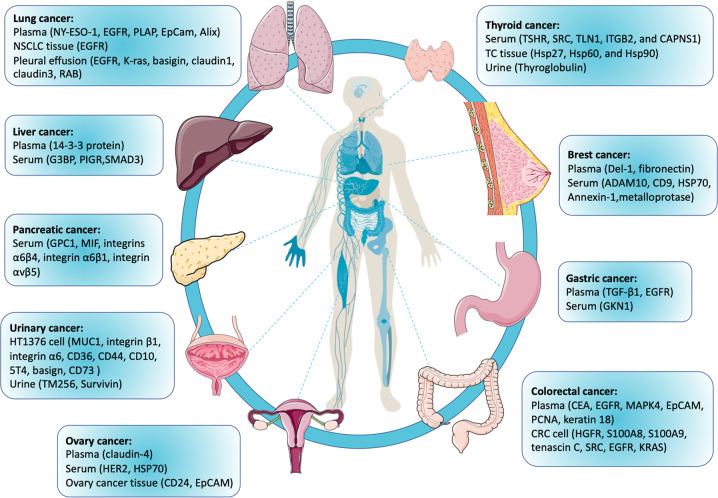
Exosomal protein biomarkers for multiple types of human cancer. Exosomal proteins obtained from body fluids (plasma, serum, urine, and pleural effusion), tumor tissue, and cultured cell lines have been reported as specific diagnostic and prognostic factors for numerous types of cancer, including breast, urinary, lung, gastric, liver, colorectal, ovarian, thyroid, and pancreatic cancers.

Although the contents of exosomes are constantly being redefined, the advantages and uniqueness of exosome cargo have proven their great potential as tumor markers. Proteins located on the surface or within the membrane of exosomes can also be applied as cancer biomarkers. Compared with other types of exosome cargo (DNA, mRNA, miRNA, circRNA, lncRNA, and metabolites), proteins can provide abundant, stable, sensitive, and distinct information. Exosomal proteins were reported as specific diagnostic and prognostic factors for numerous types of cancer, including breast, urinary, lung, gastric, liver, colorectal, ovarian, thyroid, and pancreatic cancers (Fig. 2).

### Breast cancer

Worldwide, breast cancer has become the most common cancer among women. Moreover, nearly 50% of breast cancer patients develop metastasis even after receiving systemic therapy, and these patients with metastatic disease have a 5-year survival rate of only approximately 20%^83^. Research has proven that ADAM10, metalloprotease, CD9, Annexin‐1, and HSP70 are enriched in exosomes isolated from the pleural effusion or serum of breast cancer patients^84^. Melo et al.^85^ found that 75% of breast cancer patients had a higher level of surface exosomal GPC1 expression (GPC1+) than the healthy controls. The diagnostic value of fibronectin and developmental endothelial locus‐1 (Del‐1) in breast cancer cell-derived exosomes was suggested by Moon et al.^86^, with an AUC of 0.961, a sensitivity of 94.70%, and a specificity of 86.36%^87^. Moreover, the distinct expression pattern of exosomal survivin‐2B in serum is considered a sign of early-stage breast cancer^88^. When compared with controls who were disease-free for 5 years, these patients showed a dramatic elevation in the serum exosomal survivin-2B level. However, studies are required to illustrate whether exosomal proteins can be used as noninvasive and effective diagnostic markers of breast cancer.

### Urinary cancer

Numerous studies have focused on the protein components of bladder and prostate cancer cell-derived exosomes. Wang et al.^89^ conducted a proteomics study on exosomes derived from HT1376 bladder cancer cells. A total of 353 exosomal proteins were detected by MS, and elevated expression of some exosomal surface proteins, including MUC1, integrin β1, integrin α6, CD36, CD44, CD10, 5T4, basigin, and CD73, was detected. Jeppesen et al.^90^ reported that the upregulation of several proteins (i.e., vimentin, CK2α, HDGF, annexin 2, and moesin) in bladder cancer cell-derived exosomes is correlated with an increased propensity for metastasis. Urine samples collected after digital rectal examination were reported to be enriched with exosomes released from prostate cells and therefore have a high level of prostate-specific proteins^91^. Øverbye et al.^92^ studied urinary exosomal proteins in 16 prostate cancer patients and 15 healthy controls. A total of 246 differentially expressed proteins were detected between the two cohorts, 17 of which showed sensitivities above 60% at 100% specificity, and TM256 had the highest sensitivity (94%). These studies demonstrated the value of urinary exosomes in discovering proteomic biomarkers for the noninvasive diagnosis of urinary cancer.

### Lung cancer

Exosomal proteins have shown specific diagnostic value for NSCLC. Wu et al.^93^ reported that 80% of the exosomes obtained from NSCLC biopsies were EGFR-positive, compared with 2% of those from chronic inflammatory lung tissue. Moreover, exosomes can deliver EGFR to endothelial cells to stimulate the MAPK and Akt pathways, leading to VEGF overexpression and an increase in tumor vascularity. Li et al.^94^ identified the differentially expressed proteins (including EGFR, GRB2, and SRC) in exosomes from normal bronchial epithelial cells and NSCLC cells. They noted that some proteins related to cell adhesion, ECM, and proteases were highly expressed in NSCLC cell-derived exosomes. Park et al.^95^ isolated highly purified exosomes from malignant pleural effusion of NSCLC patients and identified potential diagnostic markers, including EGFR, K-Ras, basigin, carcinoembryonic antigen-related cell adhesion molecule 6, claudin1, claudin3, and RAB family proteins. Recently, an EV array was utilized to capture exosomes from the blood of NSCLC patients, and researchers constructed a diagnostic model comprising 30 exosomal proteins^96^ that had a sensitivity and specificity of approximately 75%. Thus, EV Arrays can be used to not only determine the proteomic profile of exosomes from tumor cells but also potentially diagnose lung cancer^97^. Sandfeld et al.^98^ generated an overall survival (OS) prognostic model based on multiple exosomal protein markers and found that NY-ESO-1 was correlated with a worse OS. To conclude, a multiprotein model could classify lung cancer patients with different disease stages and histology types, and exosomal proteins have the potential as diagnostic markers for lung cancer.

### Colorectal cancer

Previous studies have identified some protein biomarkers of colorectal cancer (CRC)-derived exosomes, such as CEA, EGFR, MAPK4, PCNA, and keratin 18^99^. Specifically, there are two types of exosomes (HSA+/DR4 exosomes and HAS-/DR4 exosomes) that carry CD26, CD63, and major histocompatibility complex class molecule II (MHC II)^100^. These exosomes can contact dendritic cells and strongly potentiate immune responses by decreasing the threshold level of antigen presentation required for activation at the mucosa. Proteomic analyses of CRC cell-derived exosomes have shown the specific expression of many metastatic factors, such as hepatocyte growth factor receptor, S100A8, S100A9, and tenascin C^101–103^. Using 1-D SDS–PAGE and nano-LC–MS/MS analysis, Alin et al.^104^ identified several exosomal proteins from the ascites of three CRC patients and found that some of these proteins may play roles in tumor development by disrupting epithelial polarity and affecting metastasis, proliferation, immune regulation, and angiogenesis. More recently, a phosphoproteomic analysis of metastatic CRC cell-derived exosomes showed significantly higher levels of phosphorylated proteins than did nonmetastatic CRC cell-derived exosomes, indicating that exosomes might also transfer phosphorylated proteins to target cells^105^. Thus, more research should be performed to explore the potential application of exosomal proteins in CRC diagnosis and treatment.

### Gastric cancer

Some exosomal proteins are involved in the development of GC. TGF-β1, an immunosuppressive cytokine, was detected in exosomes obtained from the serum of GC patients, and TGF-β1 levels were related to the lymphatic metastasis of GC^106^. Exosomal TGF-β1 can induce the differentiation of regulatory T cells, helping GC cells disrupt normal immune activity in the host. EGFR was found in GC cell-derived exosomes and shown to be delivered to the liver and fuse with the membrane of hepatic stromal cells^107^. EGFR then suppressed the expression of miR-26a/b and further increased the expression of hepatocyte growth factor in hepatic stromal cells^10^, promoting the development of a suitable environment for the liver metastasis of GC cells. Yoon et al.^108^ reported that serum GKN1 levels were significantly higher in healthy controls (median: 6.34 ng/μL, interquartile range (IQR): 5.66–7.54 ng/μL) than in GC patients (median: 3.48 ng/μL, IQR: 2.90–4.11 ng/μL; *P* < 0.0001). The sensitivity and specificity were 91.2% and 96.0%, respectively, for GC. Furthermore, serum GKN1 levels were shown to distinguish patients with GC from patients with colorectal, liver, lung, breast, pancreatic, ovarian, and prostatic cancer with AUC values greater than 0.94, indicating its value as a GC-specific diagnostic biomarker^109^. These studies suggest that exosomal proteins are potential diagnostic and prognostic markers for GC.

### Liver cancer

Because liver cancer commonly has nonspecific manifestations in the early stage, patients often miss the best opportunity for treatment. Thus, early diagnosis is the most important element for successful liver cancer therapy. In clinical studies, tumor markers (such as α-fetoprotein (AFP)), imaging, and histopathological biopsies are commonly used diagnostic tools for hepatocellular carcinoma (HCC). Approximately 80% of patients with primary liver cancer have elevated levels of AFP in their blood. However, approximately 50% of HCC patients are AFP-negative, so AFP has low sensitivity and specificity for HCC screening. Arbelaiz et al.^110^ evaluated the protein levels in exosomes from the serum of HCC patients and a healthy cohort and noted that G3BP and PIGR levels were dramatically increased in HCC patients; moreover, the prediction efficacy of these two exosomal proteins for HCC was higher than that of AFP. In contrast to healthy people and patients with cholangiocarcinoma, HCC patients have significantly higher levels of exosomal G3BP. The AUC was 0.904 for HCC versus control, which is higher than that for the widely used marker AFP, and 0.894 for intrahepatic cholangiocarcinoma versus HCC. Therefore, exosomal G3BP could indicate the occurrence of HCC and discriminate HCC from other hepatic diseases^111^. Fu et al.^112^ reported that HCC cell-derived exosomes contain SMAD3 protein, which could facilitate the adhesion of HCC cells. The number of SMAD3-positive exosomes was positively associated with HCC stage and pathological grade and was negatively associated with the postsurgical disease-free survival of HCC patients. Wang et al.^113^ reported that 14-3-3 protein levels were increased in HCC cell-derived exosomes and that 14-3-3 protein could impair antitumor activity through T-cell exhaustion. This group also reported that high 14-3-3 protein levels were related to larger tumor size, poorer tumor differentiation, and more advanced TNM stage. Although several studies have investigated the relationship between exosomal proteins and liver cancer, there remains insufficient in-depth research on the mechanism by which exosomes impact liver cancer formation.

### Ovarian cancer

Although serum CA125 has been widely applied as a biomarker for OC, not all OC patients have elevated CA125 levels^114^. Elevated CA125 is also detected in patients with other malignant and benign diseases. In a study of approximately 70,000 women, the CA125 test and transvaginal ultrasound did not decrease the mortality rate but resulted in unnecessary surgery because of false-positive results^115^. Therefore, it is necessary to search for novel diagnostic markers for early diagnosis. A study by Runz et al.^116^ found that CD24 and EpCAM are exosomal cargo of ovarian carcinoma cell lines and malignant ascites. Exosome-mediated proteolytic activity in the TME may promote tumor invasion into the stroma. Claudin 4-positive exosomes were detected in the blood from 32/63 OC patients but only 1/50 healthy controls, suggesting their potential as a highly sensitive and specific indicator of OC^117^. HSP70, an exosomal surface marker, is highly expressed in OC cell-derived exosomes compared with those derived from healthy controls^118^. Moreover, exosomal detection using magnetic nanobeads revealed that the serum of OC patients contained a large number of exosomes expressing HER2^119^. Further analyses are required to characterize and distinguish the populations of exosomes and explore the biological functions of exosomal proteins in vivo.

### Thyroid cancer

The diagnosis of thyroid cancer (TC) is challenging. At present, ultrasonography and fine needle aspiration cytology are widely applied for the diagnosis of TC but are not ideal, as indeterminate samples can lead to unnecessary surgeries or missed diagnoses^120^. Luo D et al.^121^ found that the levels of some exosomal proteins (SRC, TLN1, ITGB2, and CAPNS1) were associated with EMT in TC patients with lymph node metastasis. Caruso et al.^122^ reported that HSP protein levels were elevated in TC tissue compared with para-tumor thyroid tissue and benign goiters. The levels of exosomal HSP proteins were dramatically higher in presurgical TC patients than in patients with benign goiters or in TC patients after surgery^123^. TC patients usually receive thyroidectomy along with radioactive I-131 therapy, followed by thyroid ultrasonography and serum thyroglobulin (Tg) tests^124^. Huang et al. detected trends in serum Tg and urinary exosomal Tg concentrations in 16 patients with postablative TC^125^. Serum Tg was not detected in 5 patients after thyroidectomy and radioactive I-131 therapy, while urinary exosomal Tg presented an increasing trend, indicating the possible recurrence of TC. These results suggest that the application of exosomal proteins could greatly contribute to the diagnosis and clinical monitoring of TC.

### Pancreatic cancer

Exosomal proteins also have potential application value for PC diagnostics. Buscail et al. observed that GPC1 was enriched in PC cell-derived exosomes^126^. GPC1-positive exosomes showed great accuracy, with an AUC of 1.0, a sensitivity of 100%, and a specificity of 100%. In contrast, CA 19–9 was inferior at distinguishing PC patients and healthy controls (AUC of 0.739), suggesting that the sensitivity and specificity of exosomal GPC1 in diagnosing PC were higher than those of CA 19–9^127^. The abundance of GPC1-positive exosomes was correlated with tumor burden and the OS of PC patients^128^. MIFs were overexpressed in PC cell-derived exosomes, and their suppressive activity can promote the occurrence and metastasis of PC. Compared with patients without PC progression, metastatic PC patients showed a dramatic increase in MIFs, suggesting that exosomal MIFs play essential roles in metastasis and might be predictive markers for liver metastasis. A more recent study reported that the exosomal integrins α6β4 and α6β1 were correlated with the lung metastasis of PC, while integrin αvβ5 was associated with liver metastasis^129^. Thus, it is important to study the role of exosomal proteins in PC.

## The therapeutic value of exosomal proteins in human cancer

**Fig. 3:**
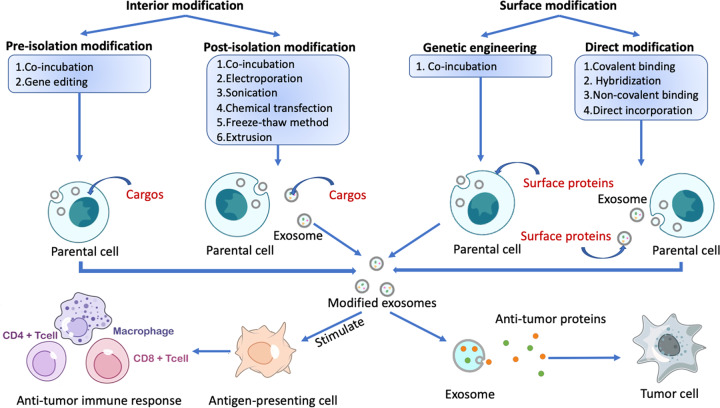
The internal and surface protein modification of exosomes. Exosomes can be modified by targeting internal proteins (adding protein cargo into the parent cell or exosome) and altering the surface (adding proteins onto the membrane of the parent cell or exosomes). Exosomes containing tumor antigens can stimulate antigen‐presenting cells (APCs) and drive antitumor immune responses in the human body. Engineered exosomes can also directly release antitumor proteins and attack tumor cells.

Before the discovery that exosomes are involved in intercellular communication, exosomes were thought to play roles in cancer therapy. It is difficult to explore the impact of exosome-mediate transport on drug delivery because the identification and isolation of specific subpopulations remain challenging^130^. Therefore, exosomes were further engineered to carry targeting ligands and stimulus-responsive factors. Exosomes can be modified in two different ways, by internal modification (in which the protein cargo within the exosome is modified) and by surface modification (in which the exosomal surface proteins are modified) (Fig. 3). According to timing, the types of modification can be further divided into preisolation and postisolation modification. Preisolation modification is performed before exosomes are isolated from cells^131^. The parental cells are modified by incubation with the desired therapeutic agents or by gene editing, leading to the encapsulation of therapeutic agents or proteins by the exosomes. In postisolation modification, drugs and therapeutic agents are directly encapsulated into exosomes, which has greater efficiency^132^. This process involves the coincubation of exosomes and therapeutic agents that can diffuse into the lumen of exosomes along the concentration gradient or the tentative disruption of the exosome membrane to allow the cargo to cross.

Engineering exosomes that contain desired proteins to prevent tumor progression is a potential application of exosomes in cancer therapy (Fig. 3)^133^. A study reported enhanced tumor targeting and antitumor activity by engineered exosomes carrying doxorubicin^134^. In another study, the parent cells of exosomes were engineered to express Lamp2b fused to αv integrin-specific iRGD peptides, which have sufficient tumor targeting properties in prostate cancer, breast cancer, cervical cancer, and PC models^135^. A subtype of MVBs called arrestin domain-containing protein 1–mediated microvesicles (ARMMs) was reported to transfer NOTCH receptors to target cells and stimulate downstream gene expression^136^. Using chimeric proteins that drive the secretion of ARMMs, these microvesicles were shown to deliver the tumor suppressor p53 protein in vivo^137^. A new hybrid exosome method was reported by Votteler et al.^138^, who introduced the concept of enveloped protein nanocages (EPNs). By incorporating various engineered proteins, EPNs utilize membrane binding and self-assembly for their biogenesis and deliver their cargo into the cytoplasm of recipient cells^139^.

The immune functions of exosomes might make them useful as specific drug transportation tools or vaccines for cancer immunotherapy^140^. Cancer vaccines present tumor antigens to immune cells, thus activating an antitumor immune response^141^. As TD exosomes carry many tumor antigens, they are involved in antigen presentation and appear to be a possible cancer vaccine^142^. Since antigen‐presenting cell-derived exosomes are dependent on MHC, they must match the MHC haplotype. However, tumor cell-derived exosomes do not require MHC haplotype matching. Therefore, it is possible to create cell-free anticancer vaccines that do not need to be engineered for every patient. In addition, exosomes carry tumor antigens that are not specific to one type of cancer, so they may be able to protect against a variety of cancers^7^. Cell hyperthermia is known to result in elevated Hsp70 levels in both cells and exosomes^143^. Hsp70 can activate dendritic cells and monocytes and stimulate TD exosome‐mediated immune responses^144^. Moreover, Hsp70 can stimulate natural killer cells to release granzyme B, which induces the apoptosis of tumor cells^145^. As a result, HSP70 has the potential as an antigen presented on the surface of exosomes to stimulate antitumor responses. Current drugs and treatments for cancer have side effects and long-term complications, and many patients eventually develop multidrug resistance. Therefore, exosomal proteins have the potential to treat various cancers, as indicated by their antitumor effects in numerous in vitro and in vivo studies.

## Future perspective and conclusion

Recently, with the popularity of proteomics analyses, the secretion and function of exosomal proteins obtained from cell lines or body fluids have been revealed, and more attention has been given to their role in predicting tumor development. Exosomal surface proteins hold clues to the mechanisms of exosome biogenesis, secretion, protein‒protein interactions, and recipient cell targeting. Exosomal proteins play essential roles in many aspects of cancer, including EMT, ECM remodeling, angiogenesis, tumor-related immune regulation, premetastatic behavior, and therapeutic resistance.

Exosomes from body fluids are selectively enriched with characteristic proteins of cancer lesions. Using known cancer surface marker antibodies fixed on a chip, exosomal surface biomarkers were detected, indicating that the identification of exosomal surface biomarkers can provide biological information on tumors. New biomarkers will be identified, and more sensitive methods will be developed for early cancer diagnosis, prognosis, and therapy evaluation. However, the challenge of exosomal proteomic analyses compared with RNA analyses is the lack of amplification procedures for protein cargo. Moreover, to efficiently integrate the results from different research teams and translate the feasibility of using exosomes in the clinic, we should standardize the methods for exosome isolation and identification.

In summary, looking forward to improvements in technology, exosomal protein content or proteomic profiles may provide not only diagnostic and prognostic clues for cancer patients but also cancer treatment options in the future.
